# Supplementary material for: Knowledge, uptake and intention to use antibiotic post-exposure prophylaxis and meningococcal B vaccine (4CMenB) for gonorrhoea among a large, online community sample of men and gender-diverse individuals who have sex with men in the UK
Source: PLOS Glob Public Health. 2024 Dec 5;4(12):e0003807. doi: 10.1371/journal.pgph.0003807 (PMC11620361; doi:10.1371/journal.pgph.0003807)
Supplement: S2 Appendix — (DOCX) [file pgph.0003807.s002.docx]

# S2 Appendix: RiiSH 2023 participant flowchart

**
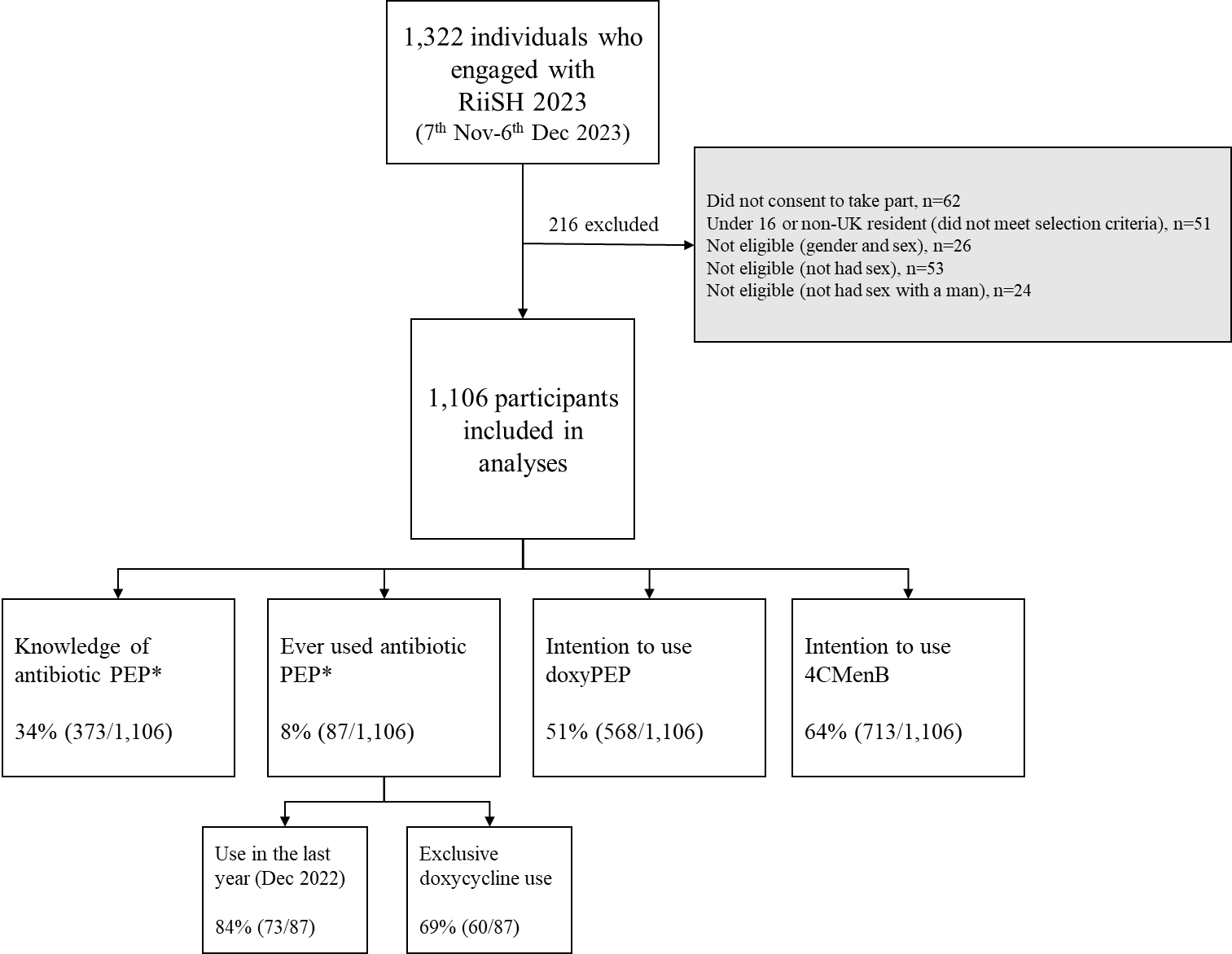
**

*See Appendix I for survey questions and preambles; report of knowledge and use of antibiotics after sex for STI prevention (i.e., antibiotic PEP). PEP=post-exposure prophylaxis. doxyPEP=doxycycline post-exposure prophylaxis.
