# Supplementary material for: Knowledge, uptake and intention to use antibiotic post-exposure prophylaxis and meningococcal B vaccine (4CMenB) for gonorrhoea among a large, online community sample of men and gender-diverse individuals who have sex with men in the UK
Source: PLOS Glob Public Health. 2024 Dec 5;4(12):e0003807. doi: 10.1371/journal.pgph.0003807 (PMC11620361; doi:10.1371/journal.pgph.0003807)
Supplement: S3 Appendix — (DOCX) [file pgph.0003807.s003.docx]

# S3 Appendix: Antibiotic regimens ever used as antibiotic post-exposure prophylaxis (PEP) among RiiSH 2023 participants

|  | n (%) |
| --- | --- |
| RiiSH participants ever reporting antibiotic PEP use* | 87 (100%) |
|  |  |
| Doxycycline only‡ | 60 (69%) |
| Azithromycin only‡ | 2 (2%) |
| Amoxicillin only‡ | 7 (8%) |
| Two or more antibiotics, including doxycycline | 8 (9%) |
| Don't know | 10 (11%) |
|  |  |
| *See Appendix I for survey questions and preambles; report of knowledge and use of antibiotics after sex for STI prevention (i.e., antibiotic PEP). ‡ Exclusive use. PEP=post-exposure prophylaxis. doxyPEP=doxycycline post-exposure prophylaxis. | |
