# Supplementary material for: Knowledge, uptake and intention to use antibiotic post-exposure prophylaxis and meningococcal B vaccine (4CMenB) for gonorrhoea among a large, online community sample of men and gender-diverse individuals who have sex with men in the UK
Source: PLOS Glob Public Health. 2024 Dec 5;4(12):e0003807. doi: 10.1371/journal.pgph.0003807 (PMC11620361; doi:10.1371/journal.pgph.0003807)
Supplement: S4 Appendix — (DOCX) [file pgph.0003807.s004.docx]

# S4 Appendix: Age and ethnic group among RiiSH 2023 participants by recruitment site

|  |  | By recruitment site | | | |
| --- | --- | --- | --- | --- | --- |
|  | Total | Facebook  (19% of sample) | Instagram  (23% of sample) | Grindr  (50% of sample) | Community-cascaded link & Twitter (8% of sample) |
|  | n (%) | n (%) | n (%) | n (%) | n (%) |
| Total | 1,106 (100%) | 213 (100%) | 253 (100%) | 553 (100%) | 87 (100%) |
|  |  |  |  |  |  |
| Median age (IQR) | 44 (34-54) | 50 (40-58) | 40 (31-49) | 44 (35-53) | 45 (35-54) |
| Age group |  |  |  |  |  |
| 16 to 24 | 68 (6%) | 7 (3%) | 26 (10%) | 30 (5%) | 5 (6%) |
| 25 to 34 | 216 (20%) | 23 (11%) | 71 (28%) | 108 (20%) | 14 (16%) |
| 35 to 44 | 283 (26%) | 47 (22%) | 63 (25%) | 150 (27%) | 23 (28%) |
| 45 to 54 | 285 (26%) | 61 (29%) | 56 (22%) | 143 (26%) | 25 (30%) |
| ≥55 | 254 (23%) | 75 (35%) | 37 (15%) | 122 (22%) | 20 (18%) |
|  |  |  |  |  |  |
| Ethnic group |  |  |  |  |  |
| White | 984 (89%) | 197 (92%) | 220 (87%) | 487 (88%) | 80 (92%) |
| All other ethnic groups | 152 (11%) | 16 (8%) | 33 (13%) | 66 (12%) | 7 (8%) |
