# Supplementary material for: Knowledge, uptake and intention to use antibiotic post-exposure prophylaxis and meningococcal B vaccine (4CMenB) for gonorrhoea among a large, online community sample of men and gender-diverse individuals who have sex with men in the UK
Source: PLOS Glob Public Health. 2024 Dec 5;4(12):e0003807. doi: 10.1371/journal.pgph.0003807 (PMC11620361; doi:10.1371/journal.pgph.0003807)
Supplement: Attachment — Submitted filename: Response to Reviewers_RiiSH2023.docx [file pgph.0003807.s005.docx]

| **Point** | **Journal requirements** | **RiiSH authors’ response** | **Action** | **Change in text** |
| --- | --- | --- | --- | --- |
| a | In your Methods section, please include additional information about your dataset and ensure that you have included a statement specifying whether the collection and analysis method complied with the terms and conditions for the source of the data. | As suggested, we have added additional text to methods section that specifies that all study methods were performed in accordance to those set out by ethnical approval group and that this was a cross-sectional analysis from the most recent round (November/December 2023) of the ‘Reducing inequalities in Sexual Health’ (RiiSH) surveys, a series of cross-sectional surveys examining the sexual health and well-being of a community sample of men and gender-diverse individuals having sex with men in the UK. | We have updated methods section with additional study detail. | **Lines 104-108 updated:**  “We ~~used data collected~~ conducted a cross-sectional study and analysis ~~from~~ of the most recent round (November/December 2023) of the ‘Reducing inequalities in Sexual Health’ (RiiSH) surveys, a series of cross-sectional surveys examining the sexual health and well-being of a community sample of men and gender-diverse individuals having sex with men ~~GBMSM~~ in the UK. Methods for RiiSH 2023 were adapted from previous study rounds (25, 26).”  **Lines 125-127 updated:**  “Ethical approval for this study was granted by the UKHSA Research and Ethics Governance Group (REGG; ref: R&D 524) and all methods were performed in accordance with guidelines and regulations set by that group.” |
| b | Please amend your detailed Financial Disclosure statement. This is published with the article. It must therefore be completed in full sentences and contain the exact wording you wish to be published.  a. Please clarify all sources of funding (financial or material support) for your study. List the grants (with grant number) or organizations (with url) that supported your study, including funding received from your institution.  b. State the initials, alongside each funding source, of each author to receive each grant.  c. State what role the funders took in the study. If the funders had no role in your study, please state: “The funders had no role in study design, data collection and analysis, decision to publish, or preparation of the manuscript.”  If you did not receive any funding for this study, please simply state: “The authors received no specific funding for this work.”" | As suggested, the authors have updated the financial disclosures statement and included the relevant organization url for reference. | We have updated financial disclosure statement. | **Lines 522-526 updated:**  “DR and CHM received funding support as part of The National Institute for Health Research Health Protection Research Unit in Blood Borne and Sexually Transmitted Infections at University College London in partnership with the UK Health Security Agency (https://bbsti.hpru.nihr.ac.uk). The funders had no role in study design, data collection and analysis, decision to publish, or preparation of the manuscript. All other authors received no specific funding for this work.” |
| c | In the online submission form you indicate that your data is not available for proprietary reasons and have provided a contact point for accessing this data. Please note that your current contact point is a co-author on this manuscript. According to our Data Policy, the contact point must not be an author on the manuscript and must be an institutional contact, ideally not an individual. Please revise your data statement to a non-author institutional point of contact, such as a data access or ethics committee, and send this to us via return email. Please also include contact information for the third party organization, and please include the full citation of where the data can be found. | Thank you. We may have incorrectly completed the online submission form for data access. If needed, we can update any online disclosures at the discretion of the editorial team.  Data will be available through reasonable request from the UK Health Security Agency (UKHSA). | As suggested, the authors have updated the data availability statement and included the relevant organization URL for reference. | **Lines 513-515 updated:**  “The data that support the findings of this study are not publicly available to protect participant privacy. However, data are available upon reasonable request from the UK Health Security Agency (UKHSA). Requests can be directed to riish_survey@ukhsa.gov.uk. ~~The data that support the findings of this study are available upon request from the UK Health Security Agency (UKHSA). Requests can be direct to Dr Hamish Mohammed (riish_survey@ukhsa.gov.uk).~~” |
| d | Please provide separate figure files in .tif or .eps format.  For more information about figure files please see our guidelines:  https://journals.plos.org/globalpublichealth/s/figures  https://journals.plos.org/globalpublichealth/s/figures#loc-file-requirements | N/A | We have uploaded Figure 1 as a .tif file. | None. |
| e | Tables should not be uploaded as individual files. Please remove these files and include the Tables in your manuscript file as editable, cell-based objects. For more information about how to format tables, see our guidelines:  https://journals.plos.org/globalpublichealth/s/tables | N/A | Tables uploaded as separate files have now been removed. All tables in the manuscript are editable, cell-based objects, therefore no changes have been made (authors are happy to resubmit in any other form as needed). | None. |
| f | Please review your reference list to ensure that it is complete and correct. If you have cited papers that have been retracted, please include the rationale for doing so in the manuscript text, or remove these references and replace them with relevant current references. Any changes to the reference list should be mentioned in the rebuttal letter that accompanies your revised manuscript. If you need to cite a retracted article, indicate the article’s retracted status in the References list and also include a citation and full reference for the retraction notice. | References have been checked and no retracted articles have been cited.  We have updated the following reference as it has now been published in a peer reviewed journal (note, the preprint was initially cited):  Baldry G, Phillips D, Wilkie R, Checchi M, Folkard K, Simmons R, et al. Factors associated with human papillomavirus, hepatitis A, hepatitis B and mpox vaccination uptake among gay, bisexual and other men who have sex with men in the UK– findings from the large community-based RiiSH-Mpox survey. International Journal of STD & AIDS. 2024:09564624241273778. | Reference updated. | **Reference section updated accordingly.**  **Other added citations are found in responses to reviewers below.** |

| **Point** | **Reviewer comment** | **RiiSH authors’ response** | **Action** | **Change in text** |
| --- | --- | --- | --- | --- |
|  | **Reviewer #1** |  |  |  |
| 1 | This is a clearly written manuscript with compelling findings. However I have several comments and suggestions regarding the generalizability of the findings to the overall target population. | Thank you for your time in reviewing and providing constructive feedback for this manuscript. | N/A | N/A |
| 2 | While the authors do mention limitations of the recruitment method, particularly the possibility of digital exclusion, this is a major concern with regard to potential generalizability of these findings. It would be helpful to provide some context for how representative this sample is of what is known about GBMSM in the UK, for example with regard to education level, race, etc. | Thank you for your comment, we acknowledge your concerns. As a whole, there are unique challenges in assessing the generalisability of survey findings in relation to the wider population of men and gender-diverse individuals having sex with men (including transgender and nonbinary individuals) in the UK given limited population-level data available.  To help clarify these challenges and contexualise our survey sample against available data, we have expanded our limitations section and introduced references probability survey data (Natsal-3) and note limitations in available census data.  We have also expanded on the findings (previously cited), that highlight the differing sexual risk behaviours among  Participants taking part in behavioural surveys and those reporting the use of internet and social networking in finding sexual partners. | In the limitations section, we have added an additional comparison and citation and expanded on limitations addressing generalisability.  Additional citations added:  (38) Mercer CH, Prah P, Field N, Tanton C, Macdowall W, Clifton S, et al. The health and well-being of men who have sex with men (MSM) in Britain: Evidence from the third National Survey of Sexual Attitudes and Lifestyles (Natsal-3). BMC Public Health. 2016;16:525.  (41) Office for National Statistics. Sexual orientation, England and Wales: Census 2021 2021 [cited 2024 01 August].  (42) Office for National Statistics. Gender identity, England and Wales: Census 2021 2021 [cited 2024 14 August]. | **Lines 420-433 updated:**  “Compared to the most recent National Survey of Sexual Attitudes and Lifestyles (Natsal-3), a national probability survey representative of Great Britain, our survey participants report higher educational attainment and levels of employment, potentially indicating higher health literacy relative to the general GBMSM population (38). ~~Our sample population~~ Participants likely experience~~s~~ higher levels of sexual risk relative to the wider population in the UK, as GBMSM taking part in targeted behavioural surveys in the UK consistently reported more sexual risk behaviours relative to nationally representative samples (39); alongside, the use of the internet and social networking in finding partners online has been associated with the report of markers of sexual risk (40). While we aimed to recruit from a broader spectrum of ~~GBMSM~~ men and gender-diverse individuals having sex with men who likely have differing sexual risk relative to clinic recruited samples, participants likely represent key populations targeted for HIV and STI prevention interventions. Aggregate census data by sexual orientation limit comparisons of our participants in respect to the wider UK population of men and gender-diverse individuals having sex with men in the UK (41, 42).” |
| 3 | Additionally, were demographics of the participants recruited through the community-cascaded link different from the remainder of the sample? | Overall, participants recruited through the community-cascaded link were broadly representative of all participants by age and ethnic group, however, this is subject to high variability given small sample size (representing 7% of total recruitment).  We do see some variation in Facebook recruited participants (e.g. older, less ethnically diverse) and for transparency, have added an additional Appendix table to highlight age and ethnic group distributions by recruitment site. However, to adhere to UKHSA HIV and STI Data Publication Policy we have aggregated ethnic groups and those recruited from the community-cascaded link and Twitter to minimise small cell count and to reduce the risk of deductive disclosure of survey participants. | Appendix table added to supplementary material.  **Appendix table added:**  Appendix IV: Age and ethnic group among RiiSH 2023 participants by recruitment site | **Appendix IV added to supplementary materials file.** |
| 4 | In addition to concerns about AMR mentioned in the discussion, are there other potential reasons why doxyPEP ITU may be lower than 4CMenB ITU? Eg, concerns about side effects, need for adherence to dosing/timing recommendations, etc. | Within our discussion (lines 397-406, we outlined a series of potential reasons that may explain lower doxyPEP ITU relative to 4CMenB ITU.  These include AMR concerns given extensive messaging around antimicrobial stewardship in the UK. We found few studies exploring preferences in these novel STI prevention interventions but cite qualitative findings suggesting wider acceptability of vaccination given concerns about side effects and safety (we have highlighted this further our manuscript). Alongside, high vaccination uptake of opportunistically offered vaccines in sexual health services could facilitate greater acceptability of 4CMenB.  Given limited literature exploring views on use of either intervention, we suggest further studies investigating preferred prevention interventions among men and gender-diverse individuals having sex with men. | We have added additional updates to the discussion and implications section. | **Lines 400-403 updated:**  “Similar views were found in a qualitative study of SHS attendees, where there was greater acceptability and support of vaccinations for STI prevention as alternatives to antibiotic prophylaxis use given concerns about side effects and safety (33).”  **Lines 473-476 updated:**  “As we found few studies exploring the acceptability of both doxyPEP or 4CMenB among men and gender-diverse individuals having sex with men, we suggest further studies investigating preferred prevention interventions to identify knowledge gaps and health concerns that may influence uptake.”  **See related response to Point 30.**  **See related response to Point 9.** |
| 5 | This manuscript is understandably heavily focused on the UK, which does have a unique national health system that may influence both the findings and their immedicate implications. However are there implications of these findings that can be extrapolated to doxyPEP and 4CMenB public health messaging and programming outside the UK? This would be helpful for readers of a global public health journal. | Thank you. In addition to implications points as part of point 4, have added additional implications considerations for settings outside of the UK where implementation of doxyPEP and 4CMenB may not be available through freely accessible or specialist services. | We have added additional detail to implications section. | **Lines 473-480 updated:**  “While implementation of both doxyPEP and 4CMenB vaccination would likely be through free and confidential SHS in the UK, this may not be the case in other countries. Given the considerable interest in doxyPEP and 4CMenB in the UK, packaging education and outreach that includes a range of preventative interventions will be key in optimising sexual health related contacts and ensuring informed use in key populations.” |
| 6 | Lines 107-108 “online click-throughs were not captured” – please explain this for readers not familiar with data collection through online surveys | Thank you for this, we acknowledge that this may not be clear to lay readers unfamiliar with survey hosting platforms. We have expanded the explanation of this in relation to survey completion. | In the methods section, we have added additional detail to clarify. | **Lines 116-117 updated:**  “Due to survey hosting limitations, ~~online click-throughs~~ we were unable to systematically capture the number of individuals who accessed this survey without participating.” ~~were not captured.~~ |
| 7 | Lines 198-199: “reaching 68% among those with markers of sexual risk” – in the table this appears to be 58% | Thank you for identifying this discrepancy. On review, we mistakenly cited estimates for 4CMenB uptake rather than those for markers of sexual risk. We have rechecked all estimates cited in text as a result. | We have updated manuscript text to include the correct figures. | **Lines 272-273 updated:**  Among all participants, 51% (95% CI: 47%-56%, 568/1,106) reported doxyPEP ITU, reaching 58% (95% CI: 51%-67%, 211/361) ~~68% (95% CI: 60%-69%, 487/713)~~ among those with our composite marker of sexual risk (Table 3.) |

| **Point** | **Reviewer comment** | **RiiSH authors’ response** | **Action** | **Change in text** |
| --- | --- | --- | --- | --- |
|  | **Reviewer #2** |  |  |  |
| 8 | In their manuscript, Ogaz et al. examine the knowledge, uptake and intention to use antibiotic post-exposure prophylaxis and meningococcal B vaccine for N. gonorrhoea infection among GBMSM in the UK. They observed that nearly half of respondents were highly likely to take up either strategy. The research question is of interest; however, there are certain issues that need to be considered. | Thank you for your time in reviewing and providing constructive feedback for this manuscript. | N/A | N/A |
| 9 | There are three outcomes studied and the link between the three are not entirely obvious. One involves the actual use of antibiotics for PEP (which is not exclusively doxyPEP). The others involve intent to use two very different preventative strategies: doxyPEP and one-time vaccination to reduce infection:  - From the theory of planned behavior, intent is the single most important driver of using a given preventative measure. Why would half of respondents have intent to use doxyPEP, but only a small proportion have ever used it? The analysis could focus on the differences in determinants of those who have intent versus actual use, given the limitation that doxyPEP is not (yet) recommended in the UK and the questions were not asked on the same prevention strategy (i.e., “any antibiotic” for use versus “doxycycline” specifically for intent).  - Alternatively, GBMSM are confronted with two prevention strategies: doxyPEP or vaccination. What are the determinants of intending to use only doxyPEP, only vaccination or both versus none? This multinomial outcome could be more helpful in planning provisions or tailoring needs.  I would suggest that the authors focus on one of these research questions above. | Thank you for your feedback. While we agree there is interest in 1) why those who intended to use doxyPEP did report prior STI antibiotic prophylaxis use and/or 2) why there is 4CMenB vaccination preference over doxyPEP, there is a very high degree of overlap and very strong positive correlation between intention to use doxyPEP and doxyPEP – as a result, there are insufficient observations to perform a multinomial regression.  Moreover, while the proposed question is of interest, it was not one of our a priori hypotheses and this additional analysis may be subject to Type I error. However, we acknowledge the importance of these insights to planning provision, our cross-sectional analysis would be limited in these assessments given the high overlap of doxyPEP and 4CMenB ITU that would likely limit analysis power and resultant inferences.  By way of background, the RiiSH study team work with community stakeholders to assess research questions and to obtain feedback on the survey questions to ensure the collection of relevant data to assess the sexual health and wellbeing of men and gender-diverse individuals having sex with men in the UK. For this analysis, we aimed to update estimates of the proportion of participants using STI antibiotic prophylaxis that were asked in previous rounds of RiiSH and other UK surveys (hence use of consistent question structure), and to explore the acceptability of novel STI prevention interventions that are not currently available through SHS, as implementation of both of these interventions are currently under review.  Overall, we agree that further exploration is warranted, and have added this as a discussion point. We have also taken on board these analysis suggestions to allow exploration of these questions for future surveys (i.e. aligning terminology is assessing use and intention to use) and for further qualitative studies aiming to explore novel STI prevention acceptability utilising a COM-B (capability, opportunity, motivation) framework for understanding behaviour. For now, we hope our cross-sectional analysis will provide a benchmark for these and other studies given limited evidence to date. | Additional discussion recommendation added. | **Lines 473-480 updated:**  “As we found few studies exploring the acceptability of both doxyPEP or 4CMenB among men and gender-diverse individuals having sex with men, we suggest further studies investigating preferred prevention interventions to identify knowledge gaps and health concerns that may influence uptake. While implementation of both doxyPEP and 4CMenB vaccination would likely be through free and confidential SHS in the UK, this may not be the case in other countries. Given the considerable interest in doxyPEP and 4CMenB in the UK, packaging education and outreach that includes a range of preventative interventions will be key in optimising sexual health related contacts and ensuring informed use in key populations.”  **See related response to Point 4.**  **See related response to Point 30.** |
| 10 | It also seems that transgender women were included in the population (ln 109), but this could be a mistake. This key population is completely different than GBMSM and should either be considered separately (i.e., stratified) or not included in analysis. | Thank you for your comment. We acknowledge that transgender women, nonbinary, and gender-diverse individuals assigned male at birth are key populations with differencing experiences in terms of STI/HIV outcomes, access, and related stigma compared to cisgender and transgender men who have sex with men. However, we clearly included these groups in recruitment (explicitly specified in participant information) (i.e., self-identifying men [cisgender/transgender], transgender women, or gender-diverse individuals assigned male at birth [AMAB], aged ≥16 years, resident in the UK and reporting sex with a man in the last year, noted in lines 118-123.  We have included all eligible participants from transgender and nonbinary groups (n=55) to improve visibility of gender minorities included in research and health-reporting (see Logie et al), though we do acknowledge findings may not be representative of wider groups (noted in limitations, lines 431-433).  As such, we have updated our title to reflect our entire participant population and have made terminology changes throughout the text.  Logie CH, van der Merwe LLA, Scheim A. Measuring sex, gender, and sexual orientation: one step to health equity. Lancet. 2022;400(10354):715–7. | We have updated title and terminology throughout the text. | **Title update:**  “Knowledge, uptake and intention to use antibiotic post-exposure prophylaxis and meningococcal B vaccine (4CMenB) for gonorrhoea among a large, online community sample ~~of gay, bisexual and other~~ men and gender-diverse individuals who have sex men in the UK”  **Lines 118-123 updated:**  ~~GBMSM~~ Participants eligible to take part and included in analyses were self-identifying men (cisgender/transgender), transgender women, or gender-diverse individuals assigned male at birth (AMAB), aged ≥16 years, resident in the UK and reporting sex with a man in the last year (hereafter, men and gender-diverse individuals who have sex with men). Gender identity and sex a birth was derived from responses from a two-step question (Appendix I).  **See terminology changes throughout text (i.e. GBMSM to ‘participants’). Note, we still utilise the GBMSM acronym, where applicable, for citing relevant literature.**  **See related response in Point 32.** |
| 11 | ln 32. “Emerging evidence”? There were several RCT that confirmed this. Not sure if this evidence is still emerging. | Thank you for this comment. While initial RCT analyses assessing the efficacy of doxyPEP have been outside the last 5 years (Molina et al 2018), more recently reported work has affirmed efficacy in other settings. However, we agree that the use of ‘emerging’ in this context may not be clear to readers. | We have updated the abstract text to remove ‘emerging’. | **Lines 32-33 updated:**  There is ~~emerging~~ evidence of the efficacy of doxyPEP in preventing bacterial STIs; however limited data exist on the extent of use in the UK. |
| 12 | ln 49. What were the numbers and % of individuals responding with intention? | For ease, we have added additional information in the abstract. Additional information is outlined in Table 3, 4 and Appendix II | We have updated abstract text to include further detail. | **Lines 48-49 updated:**  “Over half of participants reported doxyPEP ITU (51% [95% CI: 47%-56%], 568/1,106) while over two-thirds (64% [95% CI: 60%-69%], 713/1,106) reported 4CMenB ITU.” |
| 13 | ln 53. How are the authors defining “high levels”? | Thank you, while among GBMSM, uptake exceeding 50% would surpass that of many other HIV/STI prevention interventions in the UK (based on national surveillance), we see how this may be ambiguous to readers not familiar with UK STI/HIV prevention uptake. We have reframed this sentence. | We have updated the abstract discussion section to clarify opening sentence. | **Lines 55-57 updated:**  ~~There were high levels~~ “Findings demonstrate considerable interest in the use of novel STI prevention interventions, more so for 4CMenB vaccination relative to doxyPEP.” |
| 14 | ln 55-57. This sentence is ambiguous and cannot be concluded from the data provided. Please omit. | Thanks, while we agree that this point may not be best placed for abstract conclusion, we expand on the importance of crafting appropriate messaging surrounding these interventions in our concluding paragraph. | We have removed a sentence from the abstract section and updated the discussion. | **Lines 55-57 updated:**  ~~There were high levels~~ “Findings demonstrate considerable interest in the use of novel STI prevention interventions, more so for 4CMenB vaccination relative to doxyPEP. Fewer than one in ten ~~GBMSM~~ participants had reported ever using antibiotic PEP, with most using appropriate, evidence-based antibiotics. The use of antibiotic PEP and the report of doxyPEP ITU and 4CMenB ITU was more common among those at greater risk of STIs. ~~“Future guidelines and health promotion for 4CMenB and antibiotic PEP must be carefully crafted alongside clinical experts and community partners, given intervention complexity and risk of presenting conflicting public health messages regarding antimicrobial stewardship.”~~ |
| 15 | ln 59. “unprecedented”? Were such increases not observed in the 1970s? | While there have been increases in bacterial STI since the 1970s, resurgence within the last 2 decades have surpassed highest levels on record. We feel that inclusion of this point is accurate but have updated text to highlight more recent and continued increases in the since the early 2000s. | We have updated text in introduction. | **Lines 64-66 updated:**  There have been continued increases ~~and unprecedented~~ in bacterial sexually transmitted infections (STIs) (e.g., chlamydia, gonorrhoea, syphilis) among gay, bisexual, and other men who have sex with men (GBMSM) in the UK since the early 2000s (1, 2). |
| 16 | ln 67. Should be “selection of AMR bacteria.” | Thank you, we agree and have amended. | Updated introduction text. | **Lines 71-74 updated:**  “Concerns with widespread implementation include the potential effects on selection of antimicrobial resistant~~ce~~ ~~(AMR)~~ ~~selection~~ bacteria as well as individual sexual risk perception and resultant behavioural risk compensation (4-6).” |
| 17 | ln 69-74. Although important, it is unclear how these sentences relate to the research objective. Consider removing. | Thank you. Within this passage we aimed to highlight how responses for emergent threats and HIV have been successful, while similar prevention tools for bacterial STIs control have been less successful.  While mpox transmission and associated outcomes and stigma may be different to that for (some) bacterial STIs, we re-iterate the value of community coordinated response for all STI prevention messaging and implementation. As such, we have retained text. | None**.** | None**.** |
| 18 | ln 90. “… in preparation for potential use…” What does this mean? | Thank you. We understand that it may not be clear that we are referring to availability across SHS. We will update text. | Text in introduction section changed. | **Lines 96-100 updated:**  “However, in preparation for potential availability ~~use~~ of these novel STI interventions across SHS, we explored data from the most recent round of a serial, cross-sectional survey of men and gender-diverse individuals having sex with men in the UK to examine self-reported knowledge, uptake, and regimens used as antibiotic post-exposure prophylaxis (PEP) for STI prevention.” |
| 19 | General comments in the methods: It would be helpful to state which questions were asked and how your definitions were made. Then have a separate section for the statistical analysis. | Thank you for this. Given the number of analyses undertaken in the manuscript, we opted to present questions and definitions relevant to each analysis by (labelled) subsection to allow easy reference for readers. These sections include relevant definitions. Alongside, we have provided questions relevant to each outcome in Appendix I.  We feel that these sections are appropriately detailed and have not made any changes. | None. | None. |
| 20 | Statistical analysis. It is unclear how the multivariable model was constructed. It seems that there was an a priori selection of covariables. Then the authors added all covariables with a p-value <0.05 in univariable analysis? Was there further selection (i.e., backwards stepwise selection) to create the final model? And how were issues such as collinearity or model fit assessed? (I am guessing that collinearity was the reason why the association between markers of sexual risk and doxyPEP ITU in the multivariable model, ln 214-215). From the information provided, I cannot replicate these analyses. | Thank you for this. We acknowledge, that as written, descriptions of our modelling strategy lack enough detail for reproducibility.  For modelling analysis, we examined bivariate associations to ever using antibiotic PEP, doxyPEP ITU, and 4CMenB ITU, respectively. These included sociodemographic characteristics, clinical and behavioural characteristics, personal well-being and sexual satisfaction measures. We also considered a composite marker of sexual risk as a covariable in analyses. This marker was defined as reporting (in the last three months): ≥5 condomless anal sex partners, bacterial STI diagnosis, chemsex, and/or meeting partners at sex-on-premises venues, sex parties, or cruising locations. We used this marker in multivariable analyses to minimise collinearity in adjusted models. This composite was structured as a binary indicator (i.e., yes/no markers of sexual risk in the last three months) and comprised of individual markers of sexual risk examined in bivariate analyses. Bivariate (unadjusted) odds ratios (uORs), 95% CIs, and associated p-values derived from the likelihood ratio test (LRT) were calculated.  We next performed sequential, multivariable modelling to examine adjusted associations to ever using antibiotic PEP, doxyPEP ITU, and 4CMenB ITU, respectively Our initial multivariable model for each included inclusion of all sociodemographic characteristics with bivariate association to ever using antibiotic PEP and based on *a priori* knowledge, our composite marker of sexual risk minimise collinearity given correlation among individual markers of sexual risk. Adjustments for these sociodemographic characteristics and our composite measure of sexual risk were carried forward to subsequent models assessing associations to select clinical and behavioural characteristics (excluding individual markers of sexual risk included in [or as a subgroup of] our composite measure), personal well-being and sexual satisfaction measures with evidence of bivariate association to ever using antibiotic PEP, doxyPEP ITU, and 4CMenB ITU, respectively. Age-group was selected a priori for inclusion in all multivariable models. While ethnicity was also considered for inclusion, adjustment was not carried forward where p>0.05 to limit model sparsity given the low number of observations in subgroups outside of White ethnicities. Adjusted odds ratios (aORs), 95% CIs, and (LRT) p-values are presented.  We have restructured the methods section and results to address this for full transparency of our analysis strategy.  Footnotes also highlight additional modelling detail for readers. | We have fully restructured the methods and results sections to improve analysis transparency, reproducibility, and narrative flow.  Reference added:  Miller SJ, Harrison SE, Sanasi-Bhola K. A Scoping Review Investigating Relationships between Depression, Anxiety, and the PrEP Care Continuum in the United States. Int J Environ Res Public Health. 2021;18(21). | **Lines 140-184 updated:**  **See fully revised modelling methods section.**  **Lines 240-259; Lines 273-297; Lines 319-344 updated:**  **See revised sections of respective modelling results.**  **Reference section updated.**  **See related response to Point 24, 25, 34, 36.** |
| 21 | ln 155. I would consider using a flow diagram of participant selection for the main manuscript. | Thank you. We agree with the utility of this, and, as cited in the text, a diagram of participant flow has been provided in Appendix II. | None | None |
| 22 | ln 162. I would plainly state, “the period from August 2023 to questionnaire” as “lookback period” is somewhat ambiguous. | Thank you for this. For transparency and to re-iterate that August 2023 is the 3 month lookback period), we retained all details, but reordered text. | We have re-ordered text in results section. | **Lines 216-218 updated:**  “Since August 2023 (i.e., 3-month lookback period) ~~In the lookback period) (i.e., since August 2023)~~, one in five participants had ≥5 male condomless anal sex (CAS) partners (21% 231/1,106) and 8.7% (96/1,106) reported at least one positive bacterial STI test.” |
| 23 | ln 172. Curious if there were any questions on how these individuals procured doxycycline (formally or informally)? | Unfortunately, due to survey length, we were unable to ask about doxycycline sourcing, but have taken this on board for future RiiSH surveys. | None | None |
| 24 | Tables 2-4. It gives the covariables on which models were adjusted, but are these correct? Are these in addition to all other covariables listed in the column for the multivariable model? | Please see modelling description provided in Point 20.  For transparency, we included all covariables explored using bivariate logistic regression, including variables that comprised our composite measures of sexual risk (i.e. chemsex in last year, bacterial STI diagnosis since August 2023, meeting sex partners in sex-on-premises venue, sex party or cruising location, and or the report of ≥5 male condomless anal sex partners.  We next performed sequential, multivariable modelling to examine adjusted associations to ever using antibiotic PEP, doxyPEP ITU, and 4CMenB ITU, respectively. Our initial multivariable model for each included inclusion of all sociodemographic characteristics with bivariate association to ever using antibiotic PEP and based on a priori knowledge, our composite marker of sexual risk minimise collinearity given correlation among individual markers of sexual risk. Adjustments for these sociodemographic characteristics and our composite measure of sexual risk were carried forward to subsequent models assessing associations to select clinical and behavioural characteristics (excluding individual markers of sexual risk included in [or as a subgroup of] our composite measure), personal well-being and sexual satisfaction measures with evidence of bivariate association to ever using antibiotic PEP, doxyPEP ITU, and 4CMenB ITU, respectively.  We have restructured the methods section and results to address this for full transparency of our analysis strategy.  For ease, we have kept modelling results within one table, with appropriate footnotes to highlight multivariable adjustments. If preferred by the editorial team, we can create separate tables for relevant multivariable analyses. | We have fully restructured the methods and results sections to improve analysis transparency, reproducibility, and narrative flow. | **Lines 140-184 updated:**  **See fully revised modelling methods section.**  **Lines 240-259; Lines 273-297; Lines 319-344 updated:**  **See revised sections of respective modelling results.**  **See related response to Point 20, 25, 34, 36.** |
| 25 | ln 208. What is the “age-adjusted model”? | Please see modelling description provided in Point 20.  Thank you. This referred to the age-group variable that was carried forward to multivariable modelling.  We have restructured the methods section and results to address this for full transparency of our analysis strategy.  Footnotes also highlight additional modelling detail for readers. If preferred by the editorial team, we can create separate tables for relevant multivariable analyses. | We have fully restructured the methods and results sections to improve analysis transparency, reproducibility, and narrative flow. | **Lines 140-184 updated:**  **See fully revised modelling methods section.**  **Lines 240-259; Lines 273-297; Lines 319-344 updated:**  **See revised sections of respective modelling results.**  **See related response to Point 20, 24, 34, 36.** |
| 26 | ln 240. Was “recommended for use in the UK” a part of the question in the questionnaire? If not, please omit. | Thank you for this comment, while recommendation in the UK is inferred, it was not explicitly asked in these questions, and we agree with your point that is should be removed. We have retained ‘available for use in the UK’ as each question posits availability. | Introduction and discussion text updated to reflect removal of ‘and recommended’. | **Lines 100-102 updated:**  “To gauge community interest in these novel STI preventative interventions, we also explored the intention to use doxyPEP and 4CMenB assuming ~~availability~~, efficacy and availability ~~recommendation~~ in the UK.  **Lines 365-368 updated:**  “We show that the majority of GBMSM in our community sample would choose to access to doxyPEP and the 4CMenB vaccine were they available ~~and recommended~~ for use in the UK, and that intention to use is greater in those potentially most likely to benefit.” |
| 27 | ln 250. Is there empirical evidence that doxyPEP could drive AMR? | Thank you for this comment. It is unclear from the doxyPEP RCTs cited in text as to whether doxyPEP use is associated with the development of AMR in *Neisseria gonorrhoeae* *and Staph aureus* among users (because of small numbers of diagnoses/ small number of cultured isolates/ duration of follow up etc). Reassuringly, there is no in-vivo or in-vitro evidence of tetracycline resistance in *Chlamydia trachomatis* or *Treponema pallidum*. Long-term doxycycline use among people taking it for acne or malaria prophylaxis shows a mixed picture with no definitive answer as to whether it leads to more resistance in non-STI organisms. So although definitely an area of concern, there is currently limited empirical evidence to link doxyPEP use with increasing AMR.  We’ve conservatively specified that it is unclear whether doxyPEP could increase the risk of AMR for STIs or non-STI bacteria, and this is an area for further surveillance research. | We have updated the implications section | **Lines 464-466 updated:**  “Given limited empirical evidence (13,15), it is unclear whether doxyPEP will increase the development of AMR in STIs and non-STI bacteria. However, this is a key area of concern, and a focus for surveillance and research.” |
| 28 | ln 259. “… but this may reflect the wider availability of STI testing in the UK”. Not sure I follow this argument. | Thank you for this comment. We agree that this is not fully clear as we would like to convey that differences may arise as a result of wider availabilty of other STI preventative interventions in the UK through free and confidential sexual health services. We will expand on this statement for reader clarity. | We have updated discussion points accordingly | **Lines 386-389 updated:**  “Compared with a recent study in Germany which estimate doxyPEP uptake in around 20% of GBMSM (30), our sample estimates were lower but this may reflect the wider availability of other STI preventative interventions ~~STI testing~~ in the UK through free and confidential SHS (31, 32). Differing uptake could also reflect unavailability of doxyPEP in UK SHS.” |
| 29 | ln 287. How can we inform on the risks when we do not completely know what they are? This uncertainty might need to be highlighted. | Thank you for this comment. We agree that this risks are not yet fully clear and have made this acknowledgement in the text. | We have updated discussion section. | **Lines 416-419 updated:**  “Inclusive and accessible health promotion and patient education surrounding novel STI prevention interventions, especially considering self-sourcing of antibiotics, will be imperative to minimise knowledge and uptake inequalities and to clearly inform potential, though yet unclear, risks in balance of benefits to STI prophylaxis use.”  **Lines 464-466 updated:**  “Given limited empirical evidence (13,15), it is unclear whether doxyPEP will increase the development of AMR in STIs and non-STI bacteria. However, this is a key area of concern, and a focus for surveillance and research.” |
| 30 | ln 305-326. HIV-PrEP is a very different in many respects to doxyPEP and the comparison is difficult to make. Moreover, doxyPEP is part of the CDC guidance in the United States (not just in “some US cities.”, ln 323). It is unclear how these data will be used to plan provisions (and this can be the focus point of an entirely new manuscript). Many of the policy recommendations are already stated in the conclusion paragraph. The paragraph needs to be removed. | Thank you. Yes, we acknowledge that these are very different interventions relative to HIV-PrEP – as these are all biomedical preventative interventions, the aim of this paragraph is to reflect the learning from the implementation of HIV-PrEP in the UK and the fact that the need for HIV-PrEP was underestimated (see cited article from Sullivan, A et al).  However, this study demonstrates that there is comparable interest/potential demand (that precedes availability) across SHS, as was the case with HIV-PrEP in the UK; and this may be the case in other countries.  Alongside, we see consistent uptake related inequalities across a range of STI/HIV prevention interventions (e.g. HIV PrEP, mpox vaccination) so there will be a need to ensure equitable access to doxyPEP and 4CMenB.  We suggest that need (or potential demand in some cases) should not be underestimated and there must be proactive preparation for anticipated education, health promotion and additional service provision needs to minimise gaps in knowledge, uptake and streamlined provision. We also posit (in implications section, lines 478-481) the importance of packaging prevention interventions to maximise the impact of sexual health service contacts which we feel are readily applicable to service provision planning.  Thank you for signposting to CDC related doxyPEP guidelines – these were very near the time of our article submission, so we have now integrated this into our text. | We have updated the implications section.  Reference added:  Bachmann LH, Barbee LA, Chan P, Reno H, Workowski KA, Hoover K, et al. CDC Clinical Guidelines on the Use of Doxycycline Postexposure Prophylaxis for Bacterial Sexually Transmitted Infection Prevention, United States, 2024. MMWR Recomm Rep. 2024;73(2):1-8. | **Lines 478-481 updated:**  “Given the considerable interest in doxyPEP and 4CMenB in the UK, packaging education and outreach that includes a range of preventative interventions will be key in optimising sexual health related contacts and ensuring informed use in key populations.”  **Reference section updated.**  **See related response to Point 4.**  **See related response to Point 9.** |

| **Point** | **Reviewer comment** | **RiiSH authors’ response** | **Action** | **Change in text** |
| --- | --- | --- | --- | --- |
|  | **Reviewer #3** |  |  |  |
| 31 | Thank you to the authors for conducting this work. These estimates are highly informative for both research and policy development on novel biomedical interventions for bacterial STI preventions. I have a few comments regarding some of the language used in the article as well the clarifying some of the variables and modelling strategy. | Thank you for your time in reviewing and providing constructive feedback for this manuscript. |  |  |
| 32 | These results are presented as among “gay, bisexual, and other men who have sex with men” but the inclusion criteria states that transgender women and other gender diverse individuals were included in the study. Is it possible to include further details on recruitment in the methods section (e.g., was the survey advertised as for gbMSM and some trans women ended up participating?) and clarify the language used in the article? In addition, please clarify how gender was measured (e.g., two step method vs single question). | Thank you for your comment. For brevity in our initial manuscript, transgender women and gender-diverse individuals were included as part of our description of GBMSM (based on sex assigned at birth and self-reported sexual behaviour), however, we acknowledge that this may not be clear to readership and have updated this in our text and title. Our participant group is now described as men and gender-diverse individuals who have sex with men.  As recommended, we have added additional recruitment information and clarified eligibility to participate in the RiiSH survey. | We have updated methods section to include additional detail on survey recruitment and gender identity measures.  For transparency, we have also updated supplementary material to include gender identity and sex at birth question used in the RiiSH Survey. | **Lines 113-115 updated:**  “Advertisements hosted by participating recruitment sites directed users to the online survey. Users who met inclusion criteria were asked to take part.”  **Lines 119-123 updated:**  ~~GBMSM~~ Participants eligible to take part and included in analyses were self-identifying men (cisgender/transgender), transgender women, or gender-diverse individuals assigned male at birth (AMAB), aged ≥16 years, resident in the UK and reporting sex with a man in the last year (hereafter, men and gender-diverse individuals who have sex with men). Gender identity and sex a birth was derived from responses from a two-step question (Appendix I).”  Appendix I updated to include gender identity and sex birth questions used in the survey.  **See related response in Point 10**. |
| 33 | Similarly, I understand the need to dichotomize the sexual orientation variable for the analyses, but it would be helpful to specify in the methods that the “bisexual” category is really an “other” category, and it may be worth considering re-labelling it. | Thank you. We agree with your comment and have clarified further in the methods section and updated affected tables. | Updated methods section and tables. | **Lines 167-169 updated:**  “Due to small numbers, we grouped categories for sexual orientation (bisexual, straight, or another way), ethnicity (Black, Asian, Mixed, and other), gender minority groups (transgender and nonbinary).”  **Tables 1-3 updated:**  **“**Bisexual, straight, or another way‡” |
| 34 | Can you include more details in the methods section on how variables were selected for the multivariable modelling? Why were only sociodemographic variables that were statistically significant at p<0.05 used in the adjusted model? It appears as though only variables significant at p<0.05 in the bivariable models were then moved to an adjusted estimate, is that correct? | Thank you for this comment, we’ve aimed to explain our modelling strategies more thoroughly in revised manuscript text.  For modelling analysis, we examined bivariate associations to ever using antibiotic PEP, doxyPEP ITU, and 4CMenB ITU, respectively. These included sociodemographic characteristics, clinical and behavioural characteristics, personal well-being and sexual satisfaction measures. We also considered a composite marker of sexual risk as a covariable in analyses. This marker was defined as reporting (in the last three months): ≥5 condomless anal sex partners, bacterial STI diagnosis, chemsex, and/or meeting partners at sex-on-premises venues, sex parties, or cruising locations. We used this marker in multivariable analyses to minimise collinearity in adjusted models. This composite was structured as a binary indicator (i.e., yes/no markers of sexual risk in the last three months) and comprised of individual markers of sexual risk examined in bivariate analyses. Bivariate (unadjusted) odds ratios (uORs), 95% CIs, and associated p-values derived from the likelihood ratio test (LRT) were calculated.  We next performed sequential, multivariable modelling to examine adjusted associations to ever using antibiotic PEP, doxyPEP ITU, and 4CMenB ITU, respectively Our initial multivariable model for each included inclusion of all sociodemographic characteristics with bivariate association to ever using antibiotic PEP and based on *a priori* knowledge, our composite marker of sexual risk minimise collinearity given correlation among individual markers of sexual risk. Adjustments for these sociodemographic characteristics and our composite measure of sexual risk were carried forward to subsequent models assessing associations to select clinical and behavioural characteristics (excluding individual markers of sexual risk included in [or as a subgroup of] our composite measure), personal well-being and sexual satisfaction measures with evidence of bivariate association to ever using antibiotic PEP, doxyPEP ITU, and 4CMenB ITU, respectively. Age-group was selected a priori for inclusion in all multivariable models. While ethnicity was also considered for inclusion, adjustment was not carried forward where p>0.05 to limit model sparsity given the low number of observations in subgroups outside of White ethnicities. We limited inclusion of further sociodemographic variables that where p>0.05 to also limit overfitting and to minimise model sparsity. Adjusted odds ratios (aORs), 95% CIs, and (LRT) p-values are presented.  We have restructured the methods section and results to address this for full transparency of our analysis strategy.  Footnotes also highlight additional modelling detail for readers. | We have fully restructured the methods and results sections to improve analysis transparency, reproducibility, and narrative flow. | **Lines 140-184 updated:**  **See fully revised modelling methods section.**  **Lines 240-259; Lines 273-297; Lines 319-344 updated:**  **See revised sections of respective modelling results.**  **See related response to Point 20, 24, 25, 36.** |
| 35 | Please mention in the methods that the composite of sexual risk behaviour was a binary indicator (rather than a continuous or ordinal one). | Thank you. We have added this detail to the methods section to clarify structure. | Additional variable detail added to methods section. | **Lines 164-166 updated:**  This composite was structured as a binary indicator (i.e., yes/no markers of sexual risk in the last 3 months) and was based on previously described predictors of STI prophylaxis use (10, 12). |
| 36 | Can you clarify the modelling strategy – is this one multivariable model per outcome or one adjusted model per predictor? It appears as though these are separate, adjusted models for each exposure-outcome association, but this would be helpful to clarify in the methods. | Thank you for this comment.  For transparency, we included all variables explored using bivariate logistic regression, including indicators that comprised our composite measures of sexual risk (i.e. chemsex in last year, bacterial STI diagnosis since August 2023, meeting sex partners in sex-on-premises venue, sex party or cruising location, and or the report of ≥5 male condomless anal sex partners.  Our initial multivariable model for each included inclusion of all sociodemographic characteristics with bivariate association to ever using antibiotic PEP and based on *a priori* knowledge, our composite marker of sexual risk minimise collinearity given correlation among individual markers of sexual risk. Adjustments for these sociodemographic characteristics and our composite measure of sexual risk were carried forward to subsequent models assessing associations to select clinical and behavioural characteristics (excluding individual markers of sexual risk included in [or as a subgroup of] our composite measure), personal well-being and sexual satisfaction measures with evidence of bivariate association to ever using antibiotic PEP, doxyPEP ITU, and 4CMenB ITU, respectively. Thus, we have separate, adjusted models that between our outcome of interest and predictor of interest.  We have restructured the methods section and results to address this for full transparency of our analysis strategy. | We have fully restructured the methods and results sections to improve analysis transparency, reproducibility, and narrative flow. | **Lines 140-184 updated:**  **See fully revised modelling methods section.**  **Lines 240-259; Lines 273-297; Lines 319-344 updated:**  **See revised sections of respective modelling results.**  **See related response to Point 20, 24, 25, 34.** |

|  | **Other points noted by Authors** |  |  |  |
| --- | --- | --- | --- | --- |
| **Point** |  | **RiiSH authors’ response** | **Action** | **Change in text** |
| 37 |  | Missing detail noted in Tables 1-3 | Added missing word where appropriate. | **Tables 1-3 updated:**  Median age at survey completion (standard deviation)  Mean age at survey completion (standard deviation) |
| 38 |  | Updated header to align with terminology in manuscript (discussion header) | Changed subheading in discussion | **Line 238 updated:**  ~~Factors associated with~~ Correlates of ever using antibiotic post-exposure prophylaxis (PEP) for STI prevention |
| 39 |  | Applied consistent terminology throughout manuscript | Updated of ‘dPEP’ to ‘doxyPEP’ | 1 instance |
| 40 |  | Updated Table titles to align with reviewer comments | Table 2, 3, 4 titles updated | Table 2. Correlates of ever reporting antibiotic post-exposure prophylaxis (PEP) use for STI prevention among an online community sample of ~~GBMSM~~ men and gender-diverse individuals who have sex with men taking part in the RiiSH 2023 survey  Table 3. Correlates of reporting intention to use doxyPEP (doxyPEP ITU) among an online community sample of ~~GBMSM~~ men and gender-diverse individuals who have sex with men taking part in the RiiSH 2023 survey  Table 4. Correlates of reporting intention to use 4CmenB (4CmenB ITU) among an online community sample of ~~GBMSM~~ men and gender-diverse individuals who have sex with men taking part in the RiiSH 2023 survey |
|  |  | Updated header to align with terminology in manuscript (methods header) | Changed subheading in methods | **Line 129 updated:**  Knowledge and use of antibiotic post-exposure prophylaxis (PEP) ~~antibiotics after sex~~ for STI prevention |
